# The WHO global influenza surveillance and response system (GISRS)—A future perspective

**DOI:** 10.1111/irv.12565

**Published:** 2018-06-25

**Authors:** Alan J Hay, John W McCauley

**Affiliations:** ^1^ The Francis Crick Institute London UK

**Keywords:** GISRS, global influenza surveillance and response system, influenza, surveillance , WHO

## Abstract

In the centenary year of the devastating 1918‐19 pandemic, it seems opportune to reflect on the success of the WHO Global Influenza Surveillance and Response System (GISRS) initiated 70 years ago to provide early warning of changes in influenza viruses circulating in the global population to help mitigate the consequences of such a pandemic and maintain the efficacy of seasonal influenza vaccines. Three pandemics later and in the face of pandemic threats from highly pathogenic zoonotic infections by different influenza A subtypes, it continues to represent a model platform for global collaboration and timely sharing of viruses, reagents and information to forestall and respond to public health emergencies.

## 1 INTRODUCTION

In regard to sample and data sharing, we recognize the importance of the WHO global influenza surveillance and response system (GISRS) and Pandemic influenza preparedness (PIP) framework, as well as the global initiative on sharing all influenza data (GISAID). From the Berlin Declaration of the G20 Health Ministers, May 2017.^1^

## 2 A COMPREHENSIVE INTEGRATED GLOBAL SYSTEM

The Global Influenza Surveillance and Response System (GISRS; formerly known as the Global Influenza Surveillance Network) evolved as an integrated scientific and technical global collaboration to fulfil the objectives and activities of the Global Influenza Programme (GIP),^2^ initiated in 1947 as one of the initial programmes of the newly established World Health Organisation (WHO). Seventy years on it is as relevant today, with an estimated one billion cases of influenza annually, of which 3‐5 million are severe, causing between 290 000 and 650 000 deaths,^3^ and even more so given the constant pandemic threat from recent novel zoonotic influenza infections. While the principal objectives remain little changed, its *modus operandi* has adapted to the changing intellectual, technical and political environment.

Following introduction of the first influenza vaccines in 1942 and an appreciation of a need for vaccine components to reflect changes in the circulating viruses, the establishment of the WHO Global Influenza Surveillance Network in 1952 recognised the need to monitor changes in the viruses in relation to the impact of disease. Thus from the outset the global network encompassed surveillance of the epidemiology and impact of influenza, sharing of viruses isolated by WHO designated National Influenza Centres (NICs) with WHO Collaborating Centres on influenza (WHO CCs) for antigenic characterisation of the viruses and selection of suitable vaccine viruses, in relation to the manufacture, regulation and administration/distribution of influenza vaccines.

## 3 UNDERSTANDING INFLUENZA

Initiated at a time when there was relatively little detailed knowledge about the viruses, their structure and mechanisms of infection, their ecology or the inter‐relationships among the influenza viruses infecting humans and animals, research was a high priority, not only of methods for virus detection and analysis of their antigenic properties, but also of the fundamental virological properties of the virus. Many of the advances in our understanding of influenza were made in close association with the network—the “free” sharing of viruses and information providing a vital resource for the research community. Thus, revelation of the protein composition of the virus,^4^ the segmented nature of the virus genome and the structural (sequence) relationships between segments 5 in relation to genetic reassortment, and the antigenic relationships between animal and human influenza viruses,^6^ as the basis of pandemics,^7^, ^8^ were closely associated with the work of members of the WHO network and the WHO CCs in particular. Elucidation of the crystal structure of the haemagglutinin (HA) provided the first clear understanding at the molecular level of antigenic drift,^9^ of different receptor specificities between human and animal viruses in relation to host range restriction^10^ and the mechanism of virus entry into a cell, and more generally the mechanism of membrane fusion.^11^ Other notable spin‐offs from research on resistance and immunity to infection were the discovery of interferon^12^ and the uncovering of the peptide basis of cell‐mediated immunity.^13^

It is this scientific excellence within GISRS that has provided the intellectual authority and credibility for confidence, not only in the veracity of the biannual WHO recommendations on vaccine composition, but also in the procedures/methods, guidance and interpretive tools developed to anticipate the risk from seasonal antigenic/genetic variants and emergent zoonotic infections.

Monitoring of antiviral resistance by the network^14^ has also been closely aligned with more fundamental interests in the mechanisms of antiviral action and emergence of resistance. Thus, studies of resistance to amantadine revealed the target as the novel M2 proton channel.^15^, ^16^ The emergence of resistance of H1N1 and H3N2 viruses to amantadine between 2001 and 2006 17 and of H1N1 viruses to oseltamivir in 2007‐2008^18^ illustrated how the usefulness of antivirals can be eliminated due to adaptation/evolution of the viruses, and emphasises the importance of the continuous monitoring of antiviral susceptibility, as conducted by many NICs and the WHO CCs, and of developing alternative agents and combination therapy.

Driven by a need to know how best to combat influenza, to improve the efficacy of the vaccines and the effectiveness of vaccination, and the usefulness of antivirals to reduce the disease, GISRS has kept abreast of scientific and technical advances.^19^ Molecular diagnosis has largely replaced biological methods of virus detection in the laboratory, and next‐generation sequencing (NGS) is impacting the balance between antigenic and genetic characterisation of viruses.

## 4 2003 AND BEYOND

While the outbreak of bird flu (H5N1) in Hong Kong in 1997, and its high case‐fatality rate, caught the general public imagination and emphasised the importance of pandemic preparedness, it was the re‐emergence in 2003 of the H5N1 virus and its spread to other countries,^20^ together with the outbreaks of SARS coronavirus and zoonotic H7N7 influenza, that changed the dynamics and brought much greater prominence of influenza and exposure of GISRS. While the structure and relative responsibilities were understood well by the different components of the largely informal autonomous trust‐based system, comprising WHO CCs, NICs and ERLs (Essential Regulatory Laboratories), coordinated by WHO through GIP, the persistent threat from H5N1 called for a wider appreciation of its operation and benefits, especially as regards ability to respond in the event of a pandemic. For the first time, governments were faced with the concept of preparing a “pre‐pandemic” H5N1 vaccine and stockpiling the vaccine and antivirals for use in the event that the virus became more transmissible and caused an epidemic and possibly a pandemic. While this garnered a concerted effort among public health officials, regulators and vaccine manufacturers as well as among academic scientists towards the development of better vaccines and other antiviral therapies, it also led to much greater government scrutiny of the fairness of the global system, in particular by countries that did not use seasonal influenza vaccine and benefit tangibly from participation in and contribution to GISRS by sharing viruses.

The ensuing intergovernmental negotiations culminated in the Pandemic Influenza Preparedness Framework (PIP FW),^21^ a formal agreement between Member States (MS) to promote and improve pandemic preparedness and response, whereby the sharing of “H5N1 viruses and other influenza viruses with human pandemic potential” (IVPP) would be matched by access of all countries, in particular developing countries, to vaccines, antivirals and other benefits in the event of a pandemic. A key element was the introduction of an Influenza Virus Tracking Mechanism (IVTM) to monitor sharing of IVPP and a requirement to sign a materials transfer agreement (a SMTA‐2 defined in the PIP Framework^21^) to receive PIP Biological Material from GISRS, altering the way in which viruses are openly shared within and beyond GISRS. In addition to in‐kind contributions of vaccines, diagnostics and antivirals to be made available to WHO for deployment during a pandemic and other contributions also from academic laboratories, pharmaceutical companies make financial partnership contributions (PCs) to be used to support countries to participate in GISRS and help bolster the capacities of developing countries to build pandemic preparedness and response capabilities, thus further strengthening GISRS. While advantageous in terms of support for GISRS and pandemic preparedness, implementation of the PIP FW, overseen by the PIP Advisory Group, with administrative and legal support of the WHO PIP Secretariat, has introduced an important new element, specifically with respect to monitoring the sharing of PIP Biological Materials, into the longstanding successful organisation and operation of GISRS, coordinated by the GIP.

It will therefore be important, as emphasised by the recent review of the PIP FW,^22^ to ensure that its implementation is closely aligned and integrated with the activities and operational responsibilities of the GIP and GISRS, as envisaged in the PIP FW.^21^ Any reorganisation within WHO or changes consequent to implementation of the Nagoya Protocol of the Convention of Biological Diversity^23^ likely to impact GISRS should respect the national as well as international responsibilities of GISRS' components and maintain the fundamental structure and essential autonomy of GISRS, so as not to diminish the contribution of GISRS to global public health.

GISRS provides an excellent example of an effective early warning system with timely reporting and sharing of urgent information as well as an efficient and effective model of cooperation with industry. This reflects the unique features of influenza, the combination of annual epidemics and zoonotic threats of severe pandemics, and raises the question as to how best to leverage these advantages more generally to benefit response to other emergent (virus) diseases, as demonstrated in the responses to the outbreaks of SARS^24^, ^25^ and MERS‐CoV.^26^, ^27^ More in tune with seasonal influenza surveillance is the leveraging of GISRS for a pilot programme on RSV^28^ to develop an evidence base for informing future vaccination policies at national and global levels.

The recent expansion in the number and capacities of NICs over the past decade, with about 60% of countries participating in global influenza surveillance, has been due in part to pandemic threats from zoonotic infection (H5N1, H7N9, etc.) and the required notification under the International Health Regulations (IHR) of all human infections with novel influenza viruses, as well as the response to the 2009 pandemic. Bolstered by very active technical support in the provision of protocols and CDC diagnostic reagents for detection of seasonal and zoonotic (novel) infections, all regularly updated, and including training and associated performance evaluation, this momentum is likely to continue. Provision of guidance documents and analysis tools by GIP will continue to enhance the capabilities of laboratories to conduct epidemiological and virological surveillance and evaluate the burden of disease from influenza and how influenza might impact other diseases, essential to develop seasonal vaccination policies.^29^, ^30^ A 21% increase in countries with flu vaccine policies between 2006 and 2016, complemented by an 87% increase in global vaccine distribution between 2004 and 2013 bodes well for the future. On the other hand, sustainability is not guaranteed, especially if none of the perceived pandemic threats materialise and resources are judged to be better employed elsewhere. Maintenance of the “basic” seasonal surveillance capacity of the laboratory network by member states with NICs, independent of financial support from partnership contributions to the PIP FW or other external funding, will be essential and critical to sustain national capability to respond to seasonal epidemics and future pandemics. Surveillance of influenza as part of integrated disease surveillance is likely to become more prevalent as multiple parallel analysis systems become more cost‐effective and the sensitivity of point‐of‐care tests improve.

The timely response of GISRS to the 2009 H1N1 pandemic was commended by the Review Committee on the functioning of IHR 2005 in relation to the pandemic (H1N1) 2009,^31^ which acknowledged that it was “the first time that a worldwide laboratory initiative was well‐coordinated for an extended period of time,” but it cautioned that “the world is ill‐prepared to respond to a severe influenza pandemic.” More active participation in monitoring and understanding the impact of seasonal epidemics together with seasonal vaccine use, as envisaged by the “Global Agenda on Influenza Surveillance and Control” (2002) and the subsequent Global Action Plan on Influenza (GAP; 2006),^32^ are of crucial importance for a country to be prepared to react effectively and enact plans to assist, for example, in the distribution of vaccine and antivirals during a pandemic, possibly from stockpiles mobilised under the PIP FW.

While the focus of the increased emphasis on influenza has been on the pandemic potential of zoonotic infection and pandemic influenza, it is the year‐round surveillance of seasonal influenza by the NICs that provides the bedrock of the global system for surveillance and the ability to respond to a pandemic, as in 2009. The strong scientific base and expertise of many of the well‐established NICs reflect to a large extent their national public health responsibilities, as part of the local healthcare system, which go beyond influenza and include more general emergency response capabilities. NIC core function reflects the interdependency of diagnosis, virological surveillance, novel virus characterisation and epidemiology, outbreak preparedness and investigation, as well as applied research on improved test methodologies and medical countermeasures. The increasing role of genetics and automation in health care systems is reflected in a move away from isolation of influenza viruses to increasing reliance by NICs on gene sequencing for virus characterisation, enabling tracking of virus transmission^33^ and the detection and monitoring of antiviral resistance mutations.^14^, ^34^ The increasing and wider application of next‐generation sequencing technology for whole‐genome sequencing will further enhance understanding of the molecular genetics of virus infection and epidemiology, and enable response to outbreaks of disease.

## 5 COMMUNICATION AND DATA SHARING

An essential aspect of the network and coordinating responsibility of the WHO GIP is the effective, rapid collation and sharing of information, via FluNet (for virological data) and FluID (for epidemiological data), including virus genetic sequence data and associated information, as well as sharing of viruses and reference reagents.

GISRS also provided the backdrop for the successful establishment in 2008 of the Global Initiative on Sharing All Influenza Data (GISAID),^35^, ^36^ which has become an integral component of the collection, analysis and timely sharing of essential influenza virus data. Developed at the time of the intergovernmental deliberations on sharing H5N1 viruses, extensive discussions involving a broad range of interests, including government and public health officials, scientists and intellectual property experts, as well as GISRS and WHO, on how best to promote timely sharing of genetic sequence data (GSD) pre‐publication, resulted in the GISAID data sharing mechanism, the essence of which is the Database Access Agreement (DAA)^35^ which governs the sharing of the data in its EpiFlu™ database. The DAA enshrines a code of conduct between providers and users of data which protects the ownership of the data while making it freely available on the proviso that users acknowledge the source of the data and, as appropriate, engage with the originators of the data in the collaborative spirit of GISRS, in a manner consistent with its original intent.^37^ Its effectiveness is demonstrated by the prompt release of genetic sequences from the first cases of H7N9 zoonotic infection in China in 2013, which enabled the rapid production by synthetic biology of a candidate vaccine virus within a few weeks,^38^ and in ensuring availability of the latest data for the biannual WHO vaccine consultation meetings (VCMs).^39^ The close alignment of the principles underlying GISAID's sharing mechanism with the seven key principles for data sharing in a public health emergency enunciated recently by the Global Research Collaboration for Infectious Disease Preparedness (GloPID‐R)^40^ illustrates once again how GISRS can lead the way in implementing innovative mechanisms and procedures. GISAID, also a trust‐based system, integrates GSD with other clinical, virological and epidemiological data, and takes advantage of the latest advances in rapid gene sequencing technologies. It serves as a good example of sharing GSD in relation to public health emergencies and can perhaps serve as an example for other pathogens such as of Ebola and Zika viruses.^41^

## 6 VIRUS CHARACTERISATION AND VACCINE VIRUS SELECTION

A crucial aspect of GISRS and part of its success is meeting the strict timelines for vaccine virus selection and the manufacture and quality control of vaccine, prior to distribution, a process that takes 6‐8 months.^32^ Thus, any developments which assist interpretation of the most up‐to‐date data to anticipate potential future antigenic changes are likely to be beneficial in avoiding a “mismatch” of vaccine to circulating strains, which reduces vaccine effectiveness, as occurred with the northern hemisphere vaccine in 2014/2015^42^, ^43^ due to the “late” emergence of an antigenic variant around the time of the VCM in February 2014. This problem serves to illustrate inherent limitations in virological surveillance linked to the inflexible timelines for vaccine production. While antigenic changes can often be explained in terms of amino acid changes, identifying “genetic signatures” which can predict changes in antigenicity from the location of amino acid changes on the structure of the HA is still difficult.^44^ Anticipation of the future fitness trajectories of particular genetic variants using predictive modelling^45^ is being assessed for its usefulness in supporting candidate vaccine virus selection, especially as the increasing volume of sequence data affords better trajectories, helping to prioritise the most likely emergent variant groups for detailed antigenic characterisation and the production of candidate vaccine viruses (CVVs). While it may be possible to anticipate the emergence of some future antigenic variants,^46^ it is not yet evident whether adopting a futuristic vaccine strain would or would not be advantageous for vaccine effectiveness.

That aside, it is ironic that with all the technical advance in molecular characterisation, antigenic characterisation of H3N2 viruses has become more difficult due to changes in both the specificity and affinity of receptor binding and consequent agglutination of red blood cells from different species, in addition to changes in antigenicity.^42^, ^47^ Conclusive results on antigenic relationships are no longer guaranteed from a simple haemagglutination inhibition (HI) test and antigenic characterisation relies on complementary results from neutralisation assays.^39^ Attempts to develop alternative assays, to circumvent the idiosyncrasies of red blood cells, to measure antibody inhibition of virus binding have not as yet been generally successful.

The wider benefits that flow from access to the latest data are evident in the advanced tools and platforms available for analysing and interpreting sequence data. For example, real‐time tracking of influenza virus evolution by *nextflu* provides open access to regularly updated analyses of the latest genetic and antigenic data.^48^ FluSurver, a tool integrated on the EpiFlu^™^ database, assists interpretation of the potential impact of particular mutations on the antigenic, functional and antiviral susceptibility properties of the HA and NA and other proteins, in relation to published information, and enables tracking of the mutations over time and with respect to geographical distribution.^49^

While vaccine manufacturers have long supported production of high growth reassortants of CVVs, increasing difficulty in obtaining egg isolates has led to closer collaboration between manufacturers and WHO CCs, ERLs and reassorting laboratories to increase the number of potential CVVs, as well as to investigate the usefulness of cell culture‐derived CVVs, first used in a vaccine for the USA in 2017‐18, in part to overcome the potential impact of substrate‐selected mutations and to promote the development of cell culture‐based vaccines.

## 7 PANDEMIC THREAT FROM ZOONOTIC VIRUSES

Much has been learned about the mechanism of HA binding to the different receptors on avian/animal and human cells for various avian subtype viruses, H5N1, H5N6, H7N9, H9N2 and H10N8, isolated from infected humans, and has indicated that affinity for both types of receptors may represent an intermediate in acquisition of human‐to‐human transmissibility.^50^, ^51^ However, what changes would be required and what environment would be conducive to such changes in the HA and in other virus proteins to effect efficient human‐to‐human transmission is simply not known.^52^, ^53^ Thus, tools for influenza pandemic risk assessment (such as IRAT from CDC^54^ and TIPRA from WHO), which provide a comparative assessment of the perceived pandemic threats from zoonotic infections, in terms of severity (based on, eg the case‐fatality ratio) and likelihood (based on frequency of human infection) and the characteristics of the virus being assessed, are limited in predictive capacity. While H7N9 viruses have a capacity to transmit by respiratory droplet between ferrets,^55^ no increase in transmissibility between infected people has yet been detected, even during the largest outbreak in 2016‐2017 and the emergence of a highly pathogenic avian influenza pathotype.^56^ Nevertheless, while such a structured framework upon which to base an assessment of zoonotic viruses is imperfect, it provides a mechanism for prioritising the risks associated with specific viruses, and there can be no doubt that enhancement of collaboration between animal health and human health sectors under the One Health banner should also help to optimise the availability of useful information.

There is a clear lack of fundamental knowledge to predict the likelihood that a particular subtype could cause a pandemic. That it was another different H1N1 virus that caused the latest pandemic,^57^ and not a novel subtype different from that already circulating, emphasises the adventitious nature of the emergence of a pandemic virus. That a number of different H1N1 subtype viruses have transferred to pigs points to a predilection of the H1N1 avian viruses for mammalian transmissibility. Moreover, the recent sporadic human infections by swine “variant,” H1N1v and H3N2v, viruses,^58^ once again begs the question as to the extent to which pandemic viruses recycle.^59^ Nevertheless, the persistent circulation of the H3N2 subtype for almost 50 years attests to its resilience and ability to continue to adapt in the human population.

## 8 SCIENTIFIC INNOVATION

The pandemic threat also stimulated the WHO GIP to become more proactive and build upon the scientific ethos of GISRS and harness its expertise and that of the wider influenza research community in developing a WHO Public Health Research Agenda for influenza to focus efforts on reducing the risk of zoonotic infection and minimise its impact, as well as to improve clinical management. An update in 2017 identified priorities for the next 5‐10 years to include a better understanding of the burden of seasonal epidemic influenza and its economic consequences in different countries; investigation of factors associated with pathogenesis and clinical severity, including secondary bacterial infections; to better understand host genetic susceptibility; and to improve healthcare in major seasonal influenza outbreaks, as well as during pandemics, by more effective use of antivirals.^60^

On the one hand, GISRS has undergone a revolution with the introduction of molecular genetic techniques in recent years and will continue to transform its capabilities with increased introduction of NGS and multiplex molecular platforms, in high throughput or point‐of care formats, for rapid highly sensitive and specific diagnosis of respiratory tract pathogens. On the other hand, the strain‐specific inactivated vaccines in use have in essence changed little over the decades since their introduction more than 70 years ago. Application of Synthetic Biology has the potential to tailor improvements in the yield of high growth/yield reassortants and remove for example undesirable substrate‐selected changes from seasonal vaccine seed viruses. Given the substantial lead time and the lack of availability of vaccine ahead of the first wave of the 2009 pandemic, there is major effort to develop new “universal” vaccines and vaccination strategies to stimulate broadly reactive antibodies against conserved epitopes on surface proteins, for example M2 (M2e)^61^ the HA stalk,^62^ or NA,^63^ or cell‐mediated immunity against internal proteins,^64^, ^65^ to confer protection against a broader range of influenza A subtypes, or at least different antigenic variants within a subtype. Introduction of new vaccines will impact the work of WHO CCs and ERLs and require at the very least re‐evaluation of the correlates of immunity and alternative vaccine potency methods.

Moreover, licensure of new antivirals, for example targeting the virus polymerase, or therapeutic monoclonal antibodies targeting, for example the HA stalk,^66^, ^67^ will require the application of new phenotypic assays to complement molecular markers for resistance.

## 9 LOOKING AHEAD

Having seen substantial enhancement over the past decade, GISRS currently comprises 152 institutions, including 144 NICs in 114 countries, about 60% of WHO member states, leaving still room for expansion and improvement in the capacity of the world to combat epidemic and pandemic influenza. On past experience, WHO through its GIP can look forward with optimism over the next decade to plan for and address the challenges posed by influenza and other emergent respiratory viruses. WHO and GIP can continue to strengthen GISRS, by, for example, better integration of laboratory and epidemiological data and enhanced intersectoral and institutional collaboration, including public‐private partnerships, to maximise analyses and the usefulness of available data for risk management and informing national and global health policy.

The threat of a highly pathogenic avian influenza virus causing zoonotic infections to develop into a pandemic akin to that of 1918‐19 caught the imagination and galvanised a global response, one outcome of which was the PIP Framework, instituted to bolster fairness and transparency in the benefit of the “global system.” It is important, however, to recognise and be aware of the potential future impact of changes to a network which has been largely autonomous and self‐financed by member states for 65 years, to include more formal arrangements for receiving additional financial support from PIP partnership contributions, amounting to half of the estimated running cost of GISRS, and involving WHO, on behalf of member states, directly in formal sharing of IVPP viruses.^21^ Given the success of its 70‐year‐old Global Influenza Programme, the WHO has a crucial responsibility for ensuring that these additional resources are used to the best advantage, in a synergistic manner, to strengthen GISRS, under the umbrella of its GIP, to enable countries to meet their IHR responsibilities and promote benefit sharing initiatives, as well as for ensuring that GISRS' extensive complementary scientific expertise and collaborative ethos remain fully engaged to the benefit of global health security.

While GISRS is at the heart of any response to influenza, improvements in vaccines, including the quest for a “universal flu vaccine,” and antivirals are essential to improve the world's capability and overall effectiveness in controlling the disease. GISRS' broad vision, under the watchful eye of the WHO GIP, should continue to provide leadership in its interaction with global partners and to implement technological advances to better understand factors influencing the adaptive potential and interspecies transmissibility of the viruses and the host response to human infection. It will be essential to retain the fundamental characteristics and collaborative ethos of GISRS as it evolves under the WHO Global Influenza Programme as the front‐line defence against influenza in all its forms, and as a pre‐eminent example of an integrated global system for combatting infectious disease.
